# Supplementary material for: Learning curve and surgical outcome of robotic assisted colorectal surgery with ERAS program
Source: Sci Rep. 2022 Nov 29;12:20566. doi: 10.1038/s41598-022-24665-w (PMC9709162; doi:10.1038/s41598-022-24665-w)
Supplement: Supplementary file 1 — Supplementary Information 1. [file 41598_2022_24665_MOESM1_ESM.zip › LSC Raw data-Ñ[▒K/081/N_ñw╝╨░O▒Kñσ.pdf]

W52 / W52

臺中榮民總醫院

手術室護理記錄

[redacted]

基本資料

- 室別： 開刀房-06室，手術科別： CRS，手術類別： 常規，日期： 2020/06/19
- 進入時間： 1005 ； 麻醉開始時間： 1010 ； Time Out時間： 1045 ； 劃刀時間： 1050 ； 手術結束時間： 1409 送出時間： 1420 ；
- 麻醉方式： GE

  診斷：A COLON CA

  手術名稱：1.Laparoscopic right colectomy and anastomosis
  2.Liver biopsy only
  3.Laparoscopy

  手術局部用藥：NIL

  術前預防性抗生素：
  - 抗生素名稱一： Veterin 1GM IVD(由麻醉護理人員給予)
  - 時間： 1045
  - 上次簽章: 李思瑩

  ，追加預防性抗生素：
- 手術醫師： 蔣鋒帆，鄭紹彬，賴家鈺，黃伲霖，陳琦夫
- 麻醉醫師： VS 張逸文，麻醉醫師： R 林俞均
-
  - 刷手護士A ： 陳秋誼，時間： 1005，接替刷手一： 何佳青，時間： 1130
  - 流動護士A ： 李思瑩，時間： 1005，接替流動一： 陳秋誼，時間： 1200

皮膚狀況

A-V Shunt： 無

術前： 不完整

部位(術前)：

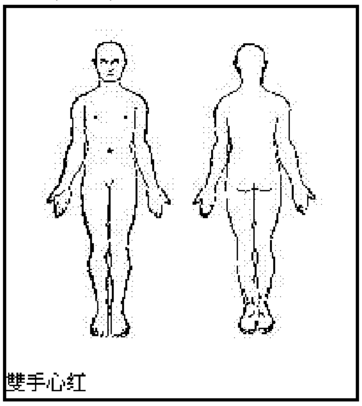

記錄者：

術後： 不完整

部位(術後)：

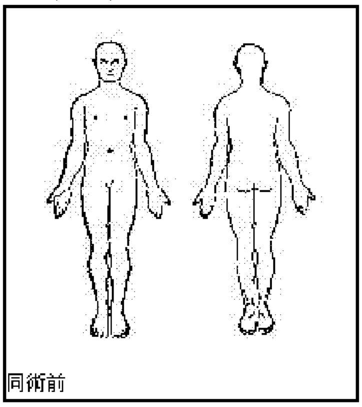

記錄者： 上次簽章: 陳秋誼

手術準備

手術臥式 單一手術臥式

-
    - 手術臥式： 仰臥
    - 使用附件： 溫毯 38 ℃，頭圈，其它： 頭架
    - 皮膚準備： 10%Aq Betadine，Hibiscrub，其它： 2% chlorhexidine
    - 電燒： 使用電燒: 電極片位置： 其它： 迴路電極墊，記錄者： 上次簽章: 李思瑩

止血帶T： 無

植入物

- 植入物名稱/號碼/數量/部位： Ethicon Endo GIA 75mm x2 /at colon

, 記錄者： 上次簽章: 陳秋誼

標本送檢

- 細菌培養 無
- 細胞學 無
- 冰凍切片 無
- 病理標本
  組織名稱一： A colon
  ,
  組織名稱二： Liver
- 截肢 無

標本交付 標本： 2 袋，標本： 0 盒，標本： 0 瓶，細胞學： 0 管，Culture Tube： 0 管，被交付醫師： R 陳瑋夫，

- 記錄者： 上次簽章: 陳秋誼
- 時間： 1410

傷口術後敷料

傷口敷料：

- 請於圖上標示部位：

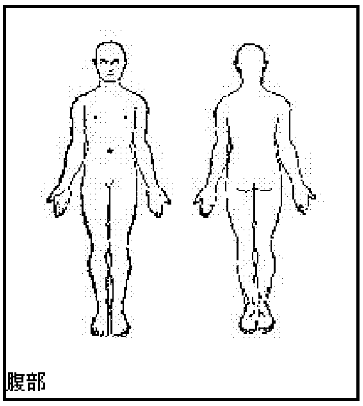

- 傷口敷料： 腹部四處傷口貼軟性敷料
  兩處取樣針傷口貼美容膠

導管

導管	天數	導管資訊	插入日期	插入時間	更換日期	拔除日期	拔除時間	拔除原因	預計拔除/更換	預計拔除/更換日期
Penrose Drain(1)	1	號碼:12 mm.; 部位:肝下;	2020/06/19	1330						
A-line (1)	1	號碼:20 G; 部位:右手; 註記:留lock;	2020/06/19	1030						
Foley(1)	1	號碼:16 Fr.; 部位:膀胱;	2020/06/19	1035						

用物計數

- 手術中出血量： 少量 c.c.

- 手術中尿量： 200 c.c.
-
    - 其它： 無

用物計數： 多切口同時縫合

- 1.器械： 正確，2.縫針： 正確，3.刀片： 正確，4.紗布： 正確，5.腹部墊： 未使用，6.紗球： 未使用，7.PEANUTS ： 未使用，8.紗條： 未使用，9.絲帶： 未使用，10.針頭： 未使用，11.Vessel loop： 未使用，12.Boots： 未使用，13.Jelco： 未使用，14.棉條： 未使用，
    - 第一次：
        - 刷手護士 ： 何佳青
        - 流動護士： 上次簽章: 陳秋誼
        - 時間： 1320

,
    - 第二次：
        - 刷手護士 ： 何佳青
        - 流動護士： 上次簽章: 陳秋誼
        - 時間： 1335

,
    - 第三次：
        - 刷手護士 ： 何佳青
        - 流動護士： 上次簽章: 陳秋誼
        - 時間： 1355

備註

備註： 腹腔鏡2號

- 記錄者： 上次簽章: 李思瑩
- 時間： 1020

填寫護理人員 陳秋誼

正式摘要

W52 / W52

doctorRank:B
